# Association Between Nocturnal Breastfeeding and Snacking Habits and the Risk of Early Childhood Caries in 18- to 23-Month-Old Japanese Children

**DOI:** 10.2188/jea.JE20140097

**Published:** 2015-02-05

**Authors:** Yoshimi Nakayama, Mitsuru Mori

**Affiliations:** 1 Hokkaido Tomakomai Public Health Center, Tomakomai, Hokkaido, Japan; 1 北海道苫小牧保健所; 2 Department of Public Health, Sapporo Medical University School of Medicine, Sapporo, Japan; 2 札幌医科大学医学部公衆衛生学講座

**Keywords:** breastfeeding, environmental tobacco smoke, early childhood caries, snacking habits

## Abstract

**Background:**

Early childhood caries (ECC) is one of the most prevalent chronic diseases among children. The aim of this cross-sectional study was to investigate the association between nocturnal breastfeeding, snacking habits, or other risk factors and ECC in 18- to 23-month-old Japanese children.

**Methods:**

Study subjects were 1675 children aged 18 to 23 months. A self-administered questionnaire was completed by parents or guardians of the children. The survey contents included such things as number of decayed, missing, and filled teeth per child, smokers in the home, nocturnal breastfeeding habit, snack times, kinds of snacks consumed ≥4 days a week, kinds of drinks consumed ≥4 days a week, parents brushing their child’s teeth daily, and the use of fluoride toothpaste. Logistic regression analysis was performed to estimate the odds of ECC.

**Results:**

The average number of decayed, missing and filled teeth was 0.10. The prevalence of dental caries was 3.3%. Nocturnal breastfeeding habits were reported in 357 subjects (21.3%). After excluding items of multicollinearity, significant associations were observed between ECC and nocturnal breastfeeding, drinking or eating sweets after dinner every day, and the intake of candy, soda and/or isotonic drinks ≥4 days a week.

**Conclusions:**

This study suggests that nocturnal breastfeeding and snacking habits are correlated with ECC.

## Abstract (ja)

**背景：:**

低年齢小児のう蝕（ECC）は、小児の間で最も罹患する慢性疾患の一つである。この研究の目的は、断面調査によって、日本の18～23ヶ月の小児の夜間授乳、間食習慣及びその他のリスク要因とう蝕との関連性を調査することである。

**方法：:**

研究対象者は、18～23ヶ月児の1,675人であった。質問調査は、保護者が回答した。調査項目は、う蝕経験歯数（dmf）、家庭での喫煙者、夜間授乳、間食時間、1週間の4日以上摂取しているおやつ及び飲み物、保護者の仕上げ磨き、フッ化物歯磨剤の使用などである。分析は、ロジスティック回帰分析を用いた。

**結果：:**

一人平均う蝕経験歯数（dmft）は、0.10本で、う蝕有病者率は3.3％であった。夜間授乳の習慣のある児は357人（21.3％）であった。多変量解析の結果、う蝕と関連のあった要因は、夜間授乳、夕食後の甘い飲み物やおやつの飲食を毎日、アメ、炭酸飲料及びスポーツドリンクの1週間に4日以上の摂取であった。

**結論：:**

この研究は、夜間授乳及び間食習慣がう蝕と関連性があることを示している。

## INTRODUCTION

Early childhood caries (ECC) is one of the most prevalent chronic diseases among children. ECC has been defined as “the presence of one or more decayed (noncavitated or cavitated lesion), missing (due to caries), or filled tooth surfaces in any primary tooth” in children from birth through 71 months of age.^1^ The etiology of ECC is multifactorial. Caries development is related to lifestyle and to several behavioral factors like poor oral hygiene and improper dietary habits. In addition, ECC has been shown to be associated with socio-economic status.^2^^–^^5^ Recently, it was suggested that children exposed to environmental tobacco smoke (ETS) also have an increased risk of dental caries in the deciduous dentition.^6^^–^^13^

Furthermore, breastfeeding has been identified as a risk factor for ECC.^13^^–^^30^ Heldeman et al^16^ conducted a cohort study in Myanmer and reported that nocturnal breastfeeding after the age of 12 months poses a risk of developing ECC. Feldens et al^20^ reported by multivariable analysis in Brazil that breastfeeding ≥7 times daily at 12 months of age was a risk factor for the occurrence of severe ECC at 4 years of age. However, the association between breastfeeding and ECC is somewhat controversial. Several studies have observed that the association between breastfeeding and ECC was insignificant,^31^^–^^36^ and the World Health Organization (WHO) has recommended that children be breastfed until 24 months of age.^37^

Previous studies^10^^,^^13^^,^^20^^,^^21^^,^^24^^,^^27^^–^^29^^,^^38^^–^^41^ have shown that snacking habits also increase the incidence of ECC. However, in most of those studies, the age of the study subjects was ≥3 years. In addition, many studies have assessed the association between the number of snack times a day or the regularity of snack times and ECC. Few studies have investigated the association between snack content in detail (eg, by including things like which kinds of drinks or snacks were consumed) and dental caries between the ages of 18 and 23 months. In our investigation of the association between detailed snack content and dental caries in 18- to 23-month-old Japanese children in the Tokachi area of Hokkaido Prefecture in 2006, snacking habits were not significantly associated with ECC.^30^

The purpose of the present cross-sectional study was to investigate the association between nocturnal breastfeeding and snacking habits, as well as other risk factors, and ECC in 18- to 23-month-old Japanese children.

## METHODS

### Subjects

The study was conducted in one city and four towns in the east Iburi region, located in the central part of Hokkaido, the northernmost island of Japan. The population of the east Iburi region was 215 233. The total number of subjects aged 18 to 23 months in the east Iburi region was 1722. Among them, 1675 (97.3%; 871 males and 804 females) received a dental examination from April 2012 to March 2013. When children in Japan reach 18 months of age, the municipality in which the family resides sponsors a physical examination that includes a dental examination, measurement of height and weight, and an interview survey with parents or guardians regarding the child’s health, in accordance with the Maternal and Child Health Act. This study was approved by the Ethical Committee of Sapporo Medical University on March 28, 2012.

### Survey method

The self-administered questionnaire was completed by parents or guardians of the children before dental examination. The questionnaire form was distributed to parents or guardians by mail beforehand and was collected on the day of the examination. After the questionnaire was completed, the data were checked by hygienists or public health nurses. We did not fill in missing values, and the data were not illogical. The dental examinations were carried out at the examination site of the municipality by 51 dentists from local dental clinics with a dental mirror under artificial light. The survey contents contained such items as: the number of decayed, missing, and filled teeth per child (dmf); whether a smoker resides in the home; the number of smokers in the home; nocturnal breastfeeding habit; bottle feeding habit; snack times; kinds of snacks (fruits or vegetables, cheese or yogurt, snack foods, ice-cream, candy, chocolate, sugar-sweetened gum, sugarless gum, pudding or jelly, Japanese crackers, bread, cake, or cookies) eaten ≥4 days a week; kinds of drinks (milk, Japanese tea or water, isotonic beverages, juice, soda, or lactic acid beverages) consumed ≥4 days a week; parents brushing their child’s teeth daily; and the use of fluoride toothpaste. We defined ETS as having at least one smoker residing in the home. Although the age in months of subjects was between 18 and 23 months, we did not obtain an accurate age in months for each individual subject. Sex was not included in the survey’s contents.

### Analyses

The outcome variable was ECC. Risk factors for the prevalence of ECC were evaluated by univariate and multivariate analysis using a logistic regression model. The odds ratios (ORs) and their 95% confidence intervals (CIs) were estimated with regard to risk factors for ECC. Before the multivariate logistic regression analysis was conducted, we evaluated multicollinearity among the variables using Spearman’s rank correlation test. Tests of statistical significance were based on a two-sided *P*-value, and the α-error was set at 5%. The SAS system (ver. 9.2; SAS Institute, Cary, NC, USA) was employed for the analysis.

## RESULTS

Figure shows distribution of study subjects according to the number of decayed, missing, and filled teeth. No caries were observed in 1620 infants. The average number of decayed, missing or filled teeth was 0.10 (standard deviation [SD] 0.65). The prevalence of dental caries was 3.3% (55/1675). Nocturnal breastfeeding habits were documented in 357 subjects (21.3%). ETS was reported for 992 children (59.2%).

**Figure.**
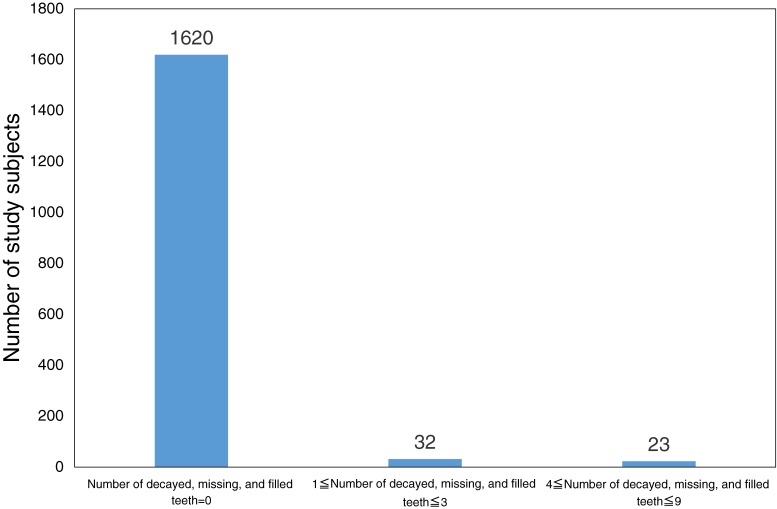
Distribution of study subjects according to the number of decayed, missing, and filled teeth

Table 1 shows crude ORs with 95% CI for ECC. Drinking or eating sweets after dinner every day (OR 3.08; 95% CI, 1.65–5.77), parents not brushing their child’s teeth at night (OR 4.33; 95% CI, 1.13–16.60), nocturnal breastfeeding (OR 3.66; 95% CI, 2.11–6.36), intake of candy ≥4 days a week (OR 3.85; 95% CI, 1.97–7.53), intake of chocolate ≥4 days a week (OR 3.68; 95% CI, 2.02–6.68), frequent intake of isotonic beverages ≥4 days a week (OR 3.28; 95% CI, 1.74–6.17), intake of soda ≥4 days a week (OR 6.14; 95% CI, 2.03–18.56), and intake of water or Japanese tea <4 days a week (OR 0.48; 95% CI, 0.24–0.98) were significantly associated with ECC. Use of fluoride agents other than toothpaste either in gel type or liquid type was not significantly associated with ECC. The existence of smokers in the home, parental smoking, maternal smoking, paternal smoking, grandparental smoking, and the number of smokers in the family were not significantly associated with ECC.

**Table 1.** Odds ratios and 95% confidence intervals of ECC using univariate logistic regression analysis

Variables	dmf ≥1 (%)	dmf = 0 (%)	OR (95% CI)
Using a fluoride toothpaste: Irregularly	35 (67.3)	1085 (68.1)	0.97 (0.54–1.74)
Drinking or eating sweets after dinner: Sometimes	11 (21.2)	339 (21.3)	1.48 (0.71–3.10)
Everyday	20 (38.5)	296 (18.6)	3.08 (1.65–5.77)
Frequency of parents brushing child’s teeth:			
Not brushing in the morning	25 (47.2)	743 (46.6)	1.12 (0.54–2.29)
Not brushing at night	3 (5.7)	23 (1.4)	4.33 (1.13–16.60)
Sometimes or not at all	11 (20.8)	246 (15.4)	1.48 (0.63–3.48)
Nocturnal breastfeeding: Yes	26 (49.1)	331 (20.8)	3.66 (2.11–6.36)
Using a bottle: Yes	11 (21.2)	195 (12.2)	1.93 (0.97–3.81)
Snack times: Irregularly	28 (54.9)	667 (43.9)	1.56 (0.89–2.73)
Consuming vegetables or fruits: ≥4 days a week	25 (47.2)	957 (59.9)	0.60 (0.35–1.04)
Consuming cheese or yogurt: ≥4 days a week	21 (39.6)	788 (49.3)	0.68 (0.39–1.18)
Consuming snack food: ≥4 days a week	33 (62.3)	827 (51.7)	1.54 (0.88–2.71)
Consuming ice cream: ≥4 days a week	12 (22.6)	223 (14.0)	1.81 (0.94–3.49)
Consuming candy: ≥4 days a week	12 (22.6)	113 (7.1)	3.85 (1.97–7.53)
Consuming chocolate: ≥4 days a week	17 (32.1)	182 (11.4)	3.68 (2.02–6.68)
Consuming cookies: ≥4 days a week	1 (1.9)	96 (6.0)	0.30 (0.04–2.20)
Consuming cake: ≥4 days a week	5 (9.4)	79 (4.9)	2.01 (0.78–5.18)
Consuming sugar-sweetened gum: ≥4 days a week	1 (1.9)	6 (0.4)	5.11 (0.61–43.19)
Consuming sugarless gum: ≥4 days a week	1 (1.9)	9 (0.6)	3.40 (0.42–27.31)
Consuming pudding or jelly: ≥4 days a week	21 (39.6)	452 (28.3)	1.67 (0.95–2.92)
Consuming Japanese crackers: ≥4 days a week	24 (45.3)	774 (48.4)	0.88 (0.51–1.53)
Consuming bread: ≥4 days a week	15 (28.3)	602 (37.7)	0.65 (0.36–1.20)
Drinking milk: ≥4 days a week	24 (45.3)	852 (53.3)	0.73 (0.42–1.26)
Drinking isotonic beverages: ≥4 days a week	14 (26.4)	158 (9.9)	3.28 (1.74–6.17)
Drinking juice: ≥4 days a week	24 (45.3)	521 (32.6)	1.71 (0.99–2.97)
Drinking soda: ≥4 days a week	4 (7.6)	21 (1.3)	6.14 (2.03–18.56)
Drinking lactic acid beverages: ≥4 days a week	4 (7.6)	179 (11.2)	0.65 (0.23–1.82)
Drinking Japanese tea or water: ≥4 days a week	43 (81.1)	1440 (89.9)	0.48 (0.24–0.98)
Drinking baby milk: ≥4 days a week	5 (9.4)	172 (10.8)	0.86 (0.34–2.20)
Smoker in family: Present	33 (63.5)	959 (60.9)	1.12 (0.63–1.98)
Smoking father: Present	28 (53.9)	826 (52.4)	1.06 (0.61–1.84)
Smoking mother: Present	14 (26.9)	274 (17.4)	1.75 (0.94–3.27)
Smoking grandparent: Present	9 (17.3)	211 (13.4)	1.35 (0.65–2.82)
Smoking parent: Present	10 (19.2)	208 (13.2)	1.57 (0.77–3.17)
Number of smokers in family: One smoker	19 (36.5)	653 (41.5)	0.95 (0.50–1.80)
Two smokers	10 (19.2)	250 (15.9)	1.30 (0.60–2.83)
Three smokers	4 (7.7)	55 (3.5)	2.36 (0.78–7.19)

CI, confidence interval; dmf, total number of decayed teeth, missing teeth or filled teeth; ECC, early childhood caries; OR, odds ratio.

As shown in Table 2, the multivariate analysis included significant variables found by univariate analysis such as drinking or eating sweets after dinner, frequency of parents brushing their child’s teeth, nocturnal breastfeeding, consumption of candy and/or chocolate, and the drinking of isotonic beverages, soda and Japanese tea or water, excluding items of multicollinearity. The multivariate analysis included 1385 infants. Drinking or eating sweets after dinner every day (OR 2.15; 95% CI, 1.00–4.62), nocturnal breastfeeding (OR 3.58; 95% CI, 1.97–6.50), intake of candy ≥4 days a week (OR 2.35; 95% CI, 1.09–5.09), intake of isotonic beverages ≥4 days a week (OR 2.20; 95% CI, 1.07–4.53), and intake of soda ≥4 days a week (OR 3.70; 95% CI, 1.07–12.81) were significantly associated with ECC.

**Table 2.** Adjusted odd ratios and 95% confidence intervals of ECC using multivariate logistic regression analysis

Variables	OR (95% CI)
Drinking or eating sweets after dinner: Sometimes	1.12 (0.51–2.50)
Everyday	2.15 (1.00–4.62)
Frequency of parents brushing child’s teeth:	
Not brushing in the morning	0.97 (0.46–2.04)
Not brushing at night	3.56 (0.87–14.60)
Sometimes or not at all	0.83 (0.33–2.07)
Nocturnal breastfeeding: Yes	3.58 (1.97–6.50)
Consuming candy: ≥4 days a week	2.35 (1.09–5.09)
Consuming chocolate: ≥4 days a week	2.05 (0.99–4.23)
Drinking isotonic beverages: ≥4 days a week	2.20 (1.07–4.53)
Drinking soda: ≥4 days a week	3.70 (1.07–12.81)
Drinking Japanese tea or water: ≥4 days a week	0.83 (0.36–1.93)

CI, confidence interval; ECC, early childhood caries; OR, odds ratio.

## DISCUSSION

The present study found that ECC was significantly associated with nocturnal breastfeeding between the ages of 18 and 23 months. Our results confirmed those of other studies showing an effect of nocturnal breastfeeding on dental caries.^15^^–^^17^^,^^19^^,^^25^^,^^26^^,^^30^ Of note, there have been several previous reports^16^^,^^19^ that the duration and frequency of breastfeeding during the day was not associated with ECC. Saliva flow decreases markedly during sleeping hours, which is thought to affect mechanical self-cleansing and the buffering capacity of saliva following fermentation of cariogenic substrates.^15^ However, subjects of several other studies showing an association between nocturnal breastfeeding and ECC were ≥2 years old.^15^^–^^17^^,^^19^ Although Mitoh^25^ and Sogabe et al^26^ investigated 18-month-old Japanese children like our study, their analysis did not adjust for confounders. We previously reported a significant multivariable-adjusted association between nocturnal breastfeeding and dental caries in 18 to 23-month-old infants in the Tokachi area of Hokkaido in 2006.^30^ Subsequently, we obtained a similar result in Iburi area of Hokkaido in this survey. Therefore, our results suggest that nocturnal breastfeeding is likely to be a risk factor for dental caries in 18- to 23-month-old Japanese children.

Breast milk has a high nutrient content and has shown numerous benefits, such as a reduced risk of otitis media, gastroenteritis, respiratory illness, sudden infant death syndrome, necrotizing enterocolitis, obesity, and hypertension.^34^ The sugar found in milk—lactose—is not fermented to the same degree as other sugars.^2^ Additionally, it may be less cariogenic because the phosphoproteins in milk inhibit enamel dissolution and the antibacterial factors in milk may interfere with growth of cariogenic oral microbial flora.^2^ Hallett et al^42^ reported that prevalence and severity of ECC decreased with an increased duration of breastfeeding up to 12 months of age compared with not breastfeeding at all.

However, in a longitudinal investigation, a significant decline over time was observed in the levels of phosphate and calcium in breast milk that help protect tooth enamel.^43^ Bowen et al^44^ reported that human milk was significantly more cariogenic than cow’s milk, probably because of its lower mineral content and higher level of lactose. High levels of lactose in human milk are rapidly fermented by cariogenic bacteria and might contribute to caries development. Thomson et al^45^ reported that human milk had a lower pH than bovine milk and bovine milk with a 2% lactose supplement, and that human milk caused greater softening of enamel than bovine milk in intra-oral tests. Therefore, nocturnal breastfeeding after the age of 12 months may pose a risk of ECC.

In multivariate analysis, frequent drinking or eating of sweets after dinner, as well as intake of candy, soda, or isotonic beverage ≥4 days a week were significantly associated with ECC. The results of this study were in accordance with several previous studies.^10^^,^^21^^,^^38^^,^^39^ Sankeshwari et al^38^ reported significant correlations between risk of ECC and sucrose exposure between meals and the total frequency of sucrose exposure. Leroy et al^10^ reported that drinking at night had a significant, positive association with visible caries in 3-year-olds in a multivariable analysis. Schluter et al^39^ reported that 4-year-old children snacking or drinking prior to bed had an increased odds ratio of fillings and/or extraction compared to those who neither snacked nor drank before bed.

However, the ages of subjects in these previous studies were ≥3 years. Our study investigated 18- to 23-month-old children. Although Kin et al^24^ investigated the association between snacking habits and ECC among 18-month-old Japanese children, the actual contents of the snacking habits were only the number of snack intake times a day and the regularity of snack times. Features of our study, which have been less common in other studies, investigated in detail the association between the snack content and risk of ECC. Although we previously investigated the association between detailed snack content and dental caries in 18- to 23-month-old Japanese children from the Tokachi area of Hokkaido in 2006, snacking habits were not significantly associated with ECC.^30^

Availability of public oral health services for children younger than 18 months may have influenced our results. In fact, one city and two towns had such service available, but two other towns in our survey area in the east Iburi region did not. Although we could not assess the extent of the impact of availability of public oral health services on children younger than 18 months in this study, the present results might suggest that oral health guidance should be performed for children younger than 18 months old, with regard to urging the avoidance of sweetened foods or beverages.

Despite findings from several previous studies suggesting that children exposed to ETS have an increased risk of dental caries in the deciduous dentition,^6^^–^^13^ ETS from the family was not significantly associated with ECC in the present study. Shenkin et al^8^ reported that American children from 4 to 7 years old residing in homes with regular smokers had a higher prevalence of caries compared to homes without regular smokers. Tanaka et al^13^ reported that ETS exposure at home was associated with an increased prevalence of dental caries among 3-year-old Japanese children. However, in most other studies, the age of subjects was ≥3 years. As the age of the present study’s subjects was between 18 and 23 months, we believe this discrepancy in findings might due to the short duration of ETS exposure.

There were some limitations to our study. First, our study did not investigate socioeconomic status, as the municipality did not allow us to collect such data. Several studies^2^^–^^5^ have reported the impact of socioeconomic status on ECC. Preschool children, especially those living in poor socioeconomic situations, are susceptible to dental caries, perhaps owing to their relatively poor nutrition, low emphasis on health behaviors, and insufficient access to dental care.^2^ In addition, Azevedo et al^46^ reported that a prolonged period of breastfeeding can be associated with a low educational level and socioeconomic status.

Second, the data on dental caries used in the present study were gathered during routine examinations by dentists at local dental clinics. The dentists were given detailed criteria for performing the examination but were not specifically trained so as to ensure standardization of their examinations.

Third, our study did not investigate the gender of children. However, previous studies^13^^,^^20^^,^^35^^,^^36^^,^^40^^,^^47^^,^^48^ have suggested that rates of caries did not differ markedly between boys and girls did.

Fourth, although the age of subjects was between 18 and 23 months, we did not obtain an accurate age in months for each individual subject. Incidence of dental caries likely increases with age, so the age in months has potential influence on the occurrence of dental caries of infants.

In conclusion, we found an association between nocturnal breastfeeding and the prevalence of dental caries among 18- to 23-month-old Japanese children after adjusting for potential confounders, such as the frequency of parents brushing their child’s teeth, the kinds of snacks eaten ≥4 days a week, and the kinds of drinks consumed ≥4 days a week. Our findings suggest that nocturnal breastfeeding and snacking habits are correlated with ECC.

## ONLINE ONLY MATERIAL

Abstract in Japanese.
