# Supplementary material for: Association Between Nocturnal Breastfeeding and Snacking Habits and the Risk of Early Childhood Caries in 18- to 23-Month-Old Japanese Children
Source: J Epidemiol. 2015 Feb 5;25(2):142–7. doi: 10.2188/jea.JE20140097 (PMC4310875; doi:10.2188/jea.JE20140097)
Supplement: Abstract in Japanese. [file je-25-142-s001.pdf]

日本の 18〜23 ヶ月の小児の夜間授乳及び間食習慣とう蝕との関連性について

中山佳美[1,2]、森満[2]

[1] 北海道苫小牧保健所

[2] 札幌医科大学医学部公衆衛生学講座

背景：低年齢小児のう蝕（ECC）は、小児の間で最も罹患する慢性疾患の一つである。この研究の目的は、断面調査によって、日本の 18〜23 ヶ月の小児の夜間授乳、間食習慣及びその他のリスク要因とう蝕との関連性を調査することである。

方法：研究対象者は、18〜23 ヶ月児の 1,675 人であった。質問調査は、保護者が回答した。調査項目は、う蝕経験歯数（dmf)、家庭での喫煙者、夜間授乳、間食時間、1 週間の 4 日以上摂取しているおやつ及び飲み物、保護者の仕上げ磨き、フッ化物歯磨剤の使用などである。分析は、ロジスティック回帰分析を用いた。

結果：一人平均う蝕経験歯数（dmft）は、0.10 本で、う蝕有病者率は 3.3%であった。夜間授乳の習慣のある児は 357 人（21.3%）であった。多変量解析の結果、う蝕と関連のあった要因は、夜間授乳、夕食後の甘い飲み物やおやつの飲食を毎日、アメ、炭酸飲料及びスポーツドリンクの 1 週間に 4 日以上の摂取であった。

結論：この研究は、夜間授乳及び間食習慣がう蝕と関連性があることを示している。

キーワード：夜間授乳、環境タバコ煙、幼児う蝕、間食習慣
